# Transcranial Magnetic Stimulation as a Diagnostic Tool in Mild Cognitive Impairment: A Systematic Review

**DOI:** 10.3390/brainsci15090969

**Published:** 2025-09-09

**Authors:** Elisa Dognini, Simona Finazzi, Elena Campana, Rosa Manenti, Maria Cotelli, Barbara Borroni

**Affiliations:** 1 Neurophysiology Unit, IRCCS Istituto Centro San Giovanni di Dio Fatebenefratelli, 25125 Brescia, Italy; edognini@fatebenefratelli.eu; 2 Department of Clinical and Experimental Sciences, University of Brescia, 25125 Brescia, Italy; simona.finazzi@unibs.it; 3 Neuropsychology Unit, IRCCS Istituto Centro San Giovanni di Dio Fatebenefratelli, 25125 Brescia, Italy; ecampana@fatebenefratelli.eu (E.C.); rmanenti@fatebenefratelli.eu (R.M.); mcotelli@fatebenefratelli.eu (M.C.); 4 Molecular Markers Laboratory, IRCCS Istituto Centro San Giovanni di Dio Fatebenefratelli, 25125 Brescia, Italy

**Keywords:** mild cognitive impairment, MCI, short latency afferent inhibition, SAI, short-interval intracortical inhibition, SICI, long-interval intracortical inhibition, LICI, intracortical facilitation, ICF, transcranial magnetic stimulation, TMS, diagnostic markers, Alzheimer disease, frontotemporal dementia

## Abstract

**Background/Objective**: Mild cognitive impairment (MCI) often represents the prodromal stage of neurodegenerative dementia. Identification of Alzheimer disease (AD) and other dementias in the MCI stage is essential for early intervention. Transcranial magnetic stimulation (TMS) has gained interest as a non-invasive method to evaluate cortical excitability and neurotransmitter function. This systematic review aims to evaluate the diagnostic utility of TMS-derived indices, such as short-latency afferent inhibition (SAI), short-interval intracortical inhibition (SICI), intracortical facilitation (ICF), and long-interval intracortical inhibition (LICI) in MCI populations. **Methods**: Following PRISMA guidelines, 14 studies were selected, encompassing 476 MCI patients. Reported outcomes related to TMS measures (SAI, SICI, ICF, LICI) were reviewed across various MCI phenotypes. **Results**: Most studies report reduced SAI, a marker of cholinergic dysfunction, in amnestic MCI and MCI due to AD. Alterations in SICI and ICF, markers of GABAergic and glutamatergic dysfunction, were more variable, mainly observed in MCI of non-AD type. LICI showed no consistent changes. One study demonstrated increased clinicians’ diagnostic confidence when TMS data were incorporated. **Conclusions**: TMS measures hold promise as a non-invasive tool for early and differential diagnosis of MCI. Further standardized and longitudinal research is needed to confirm its clinical applicability.

## 1. Introduction

Mild cognitive impairment (MCI) is a condition in which individuals demonstrate cognitive decline with minimal impact on instrumental activities of daily living [1].

MCI is considered a risk factor for developing dementia in the most common forms of neurodegenerative disorders, including Alzheimer’s disease (AD), frontotemporal dementia (FTD), and dementia with Lewy bodies (DLB) [2,3,4,5,6,7].

This condition can be classified as amnestic or non-amnestic, each affecting a single or multiple cognitive domains. Amnestic MCI (aMCI), which primarily impairs memory, is often a prodromal stage of AD. Non-amnestic MCI (na-MCI) involves other cognitive functions and may reflect different neuropathological processes [7,8].

Considering that people with MCI have an increased risk of developing dementia, early diagnosis is a crucial objective in clinical practice, as it allows differentiation between disorders and timely therapeutic intervention [9].

Biological markers such as cerebrospinal fluid (CSF) analysis and amyloid positron emission tomography (PET) are used in clinical work-up to increase diagnostic accuracy and confirm or rule out AD [10]. One of the main challenges in this field is to identify alternative diagnostic markers that maintain the same level of reliability while being more cost-effective and non-invasive.

Recently, transcranial magnetic stimulation (TMS) has gained attention as a promising non-invasive tool to enhance diagnostic accuracy and simplify differential diagnosis [11,12,13,14]. TMS, when combined with electromyography (EMG) or electroencephalography (EEG), allows for assessment of corticospinal (via EMG) and cortico–cortical or thalamo–cortical excitability (via EEG) and even to indirectly evaluate neurotransmitters imbalance of acetylcholine, glutamate, or GABA (gamma-aminobutyric acid) pathways [13].

Several studies have investigated the therapeutic role of TMS, particularly in the context of repetitive TMS (rTMS) [15,16,17]. These studies have demonstrated the potential of rTMS as a non-pharmacological treatment for alleviating symptoms of various disorders, showing promising applications in both psychiatric and neurodegenerative fields.

Recent studies have also applied TMS to provide real-time information about neural function at the level of specific cortical circuits [14,18]. Among the most investigated TMS-derived measures are short-latency afferent inhibition (SAI), an indirect marker of cholinergic circuits, Short-interval Intracortical Inhibition (SICI) and intracortical facilitation (ICF), which partially depend on GABAergic and glutamatergic circuits, and long-interval intracortical inhibition (LICI), which relies on GABA-B circuits [12,13,14,19,20,21,22]. These paradigms are indirect proxies of system-wide neurotransmission, with implications for cortical network health.

These indices are obtained by stimulating primary motor cortex (M1), and recent TMS-EEG studies have shown that M1-based protocols can provide valuable insights into cortical function beyond motor control, particularly in the context of cognitive decline. In patients with MCI, and also with AD, stimulation of M1 reveals altered TMS-evoked potentials, reduced inter-trial coherence, and disrupted functional connectivity compared to those in cognitively healthy controls [23,24,25,26]. These abnormalities correlate with memory and executive deficits, suggesting that M1 physiology reflects broader cortical dysfunction associated with disease progression.

Ziemann and colleagues [27] reviewed the literature on the effects of drugs on TMS-EEG and TMS-EMG measures, identifying neurotransmitter receptor functions associated with the above-mentioned indices.

SAI induces an inhibition of motor evoked potential (MEP) amplitude reflecting the sensorimotor integration mechanisms [18], due to median nerve stimulation prior to TMS stimulation over the contralateral primary motor cortex M1 around the time point of the N20 component [28]. This index involves thalamo–cortical and cortico–cortical inhibitory circuits [13], and, although the cholinergic system has the most important role in this dynamic, various pharmacological studies show a mediation through GABA_A_-receptors [27,29].

GABA_A_ receptors are also involved in SICI, especially at 2.5 ms [28], which represents short-lasting inhibitory postsynaptic potential in corticospinal neurons [13,21], resulting in reduced MEP amplitude [27,28,30].

Although the procedure used for ICF is very similar to the one used for SICI, ICF does not involve GABA_A_-mediated inhibitory neurotransmission. Instead, it reflects glutamatergic neurotransmission via N-methyl-D-aspartate (NMDA) receptors, representing intracortical excitatory neurotransmission [31] and leading to increased MEP amplitude [27,28,30].

Finally, the GABAergic system is also involved in LICI. However, unlike SICI, LICI predominantly involves metabotropic GABA_B_-receptors, which mediate inhibitory postsynaptic potentials and lead to a subsequent reduction in MEP amplitude [13,27,32,33].

Several studies have highlighted the potential clinical utility of these TMS parameters in the evaluation of various neurodegenerative disorders [12,14,15,17]. Reduced SAI has been observed in AD since the earliest disease stages [14,34]. Moreover, Shafiee and colleagues [35] demonstrated that Nucleus basalis of Meynert degeneration begins even before clinical MCI becomes apparent, with atrophy detectable in the initial phases of cognitive decline, suggesting a possible link between cholinergic neuronal loss and the reduction of cortical inhibition present from the onset of the disease.

Both SICI and ICF have been found to be significantly reduced in FTD [12,34,36,37]. In this form of dementia, both GABAergic and glutamatergic systems are disrupted. Loss of GABAergic neurons and reduced GABA levels contribute to behavioral disinhibition and executive dysfunction, while NMDA receptor dysfunction impairs synaptic plasticity and cognitive processing [38]. Together, these deficits likely underlie key cognitive and behavioral symptoms observed in FTD, which can thus be assessed using TMS, specifically through measures of SICI and ICF.

In DLB, Benussi and colleagues [39] found impaired SICI, ICF and SAI, meanwhile Marra [40] and Di Lazzaro [41] and colleagues reported reduced SAI, likely reflecting impaired cholinergic function [39,40,41].

Building on this evidence from established neurodegenerative conditions, it becomes relevant to explore whether these neurophysiological alterations can be identified in the earliest stages of neurodegenerative dementia, i.e., in the MCI phase. This review is timely because a substantial body of data has begun to accumulate, and MCI represents the prodromal stage that should be detected as early as possible, including through neurophysiological markers. The specific gap addressed by this manuscript is the utility of TMS parameters in this population, and the key question it seeks to explore is whether TMS can serve as a practical screening tool for early and differential diagnosis. Accordingly, the aim of this systematic review is to evaluate existing literature regarding the use of TMS in MCI populations, with a specific focus on the diagnostic value of SAI, SICI, ICF, and LICI as auxiliary tools for screening and early differential diagnosis.

## 2. Materials and Methods

### 2.1. Search Strategies and Selection of the Studies

**Figure 1:**
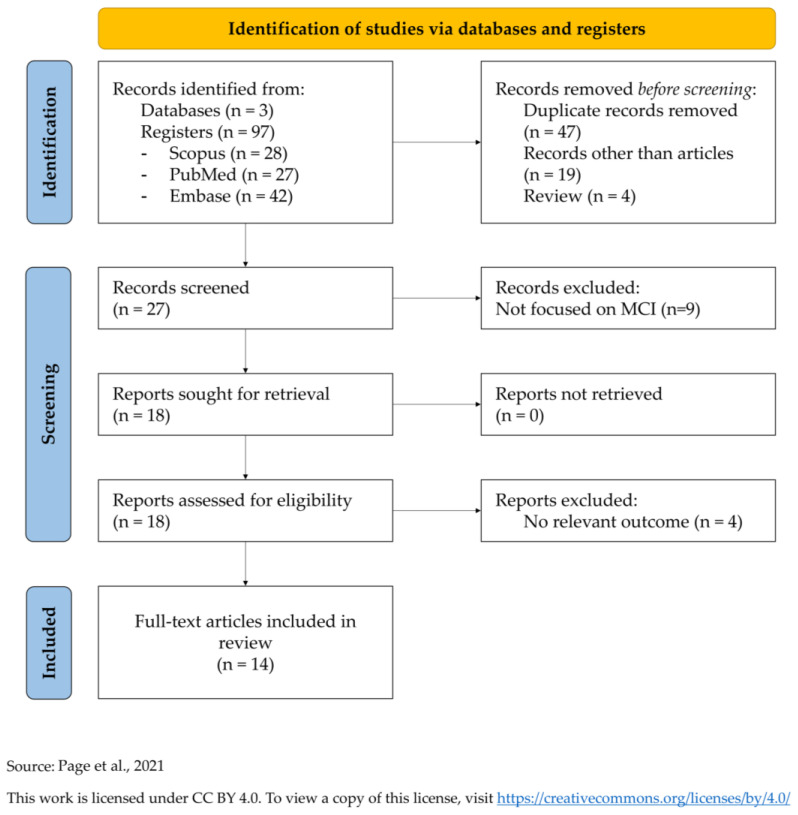
PRISMA diagram for systematic literature reviews [42] (https://www.prisma-statement.org/prisma-2020-flow-diagram; accessed on date 31 July 2025).

This systematic review was conducted in compliance with the PRISMA 2020 guidelines (Preferred Reporting Items for Systematic Reviews and Meta-Analyses) (Supplementary Materials Tables S1 and S2) [42]. The PRISMA flow diagram is shown in Figure 1.

The protocol for this review was registered in the International Prospective Register of Systematic Reviews (PROSPERO) under registration number CRD420251112713 and is available in full on the program website (https://www.crd.york.ac.uk/CRDWeb/HomePage.asp; accessed on 4 August 2025).

The electronic databases Medline (PubMed), Scopus, and Embase Web of Science Core Collection were searched for records without any time restrictions. The search strategy combined the following terms in the Title/Abstract fields: ((short-interval intracortical inhibition) OR (short-latency afferent inhibition) OR (intracortical facilitation) OR (long-interval intracortical inhibition) OR (paired pulse TMS)) AND (mild cognitive impairment).

Abstracts were reviewed and all relevant original research articles were examined in detail, including a review of the references in each publication to identify additional sources. Only English-language articles were selected (Figure 1).

### 2.2. Study Selection Criteria

Full-length articles were included if they met the following criteria: (i) original research; (ii) studies primarily focusing on at least one of the indices of interest (e.g., SAI, SICI, ICF, LICI); (iii) studies focusing on MCI or investigating MCI in the context of co-occurring neuro-degenerative disorders; and (iv) published before the 31 July 2025. Studies focusing on MCI but that did not investigate any of the TMS indices of interest were excluded.

Articles published in languages other than English, animal studies, reports of secondary data such as meta-analyses or reviews or letters were excluded.

### 2.3. Data Collection and Extraction

Two authors (E.D. and S.F.) independently removed duplicates, review articles, and conference abstracts. They then screened the remaining abstracts independently and selected articles that met the inclusion criteria. Disagreements regarding inclusion or exclusion were resolved by a third author (B.B.) through independent evaluation. Following this initial screening, the full texts of the selected articles were evenly distributed among two authors (E.D. and S.F.) for detailed review until the final corpus of literature included in this review was established. We extracted baseline information from the individual studies, including publication year, study design, participants’ characteristics and disease type. Moreover, outcome measures (SAI, SICI, ICF, LICI) were extracted.

### 2.4. Quality Assessment

All studies that met the inclusion criteria were assessed using the quality assessment ‘Quality Assessment Tool for Observational Cohort and Cross-Sectional Studies’ (https://www.nhlbi.nih.gov/health-topics/study-quality-assessment-tools; accessed on 1 August 2025) by two independent assessors (E.D. and S.F.) and disagreements were resolved through consensus or referral to a third reviewer (B.B.). Each record was classified according to the answers obtained in the quality assessment. All scores assigned to each study were agreed upon by consensus and are presented in Supplementary data (see Supplementary Materials Figure S1 for details).

## 3. Results

### 3.1. Study Characteristics

Out of 97 initial records, 70 articles were rejected before the screening and other 13 articles after the screening. Finally, 14 full-text articles were included for this systematic review. All studies are summarized in Table 1 [43,44,45,46,47,48,49,50,51,52,53,54,55,56].

Most of the studies compared the TMS indices between different groups. One study evaluated the effects of pharmacological treatment on the TMS measures [50], while one study [52] investigated the ability of TMS indices to increase diagnostic confidence (DC) and accuracy in discriminating different MCI subtypes. The fifteen included articles regarded 812 subjects, of whom 476 were MCI subjects, and were published between 2007 and 2024.

### 3.2. Short-Latency Afferent Inhibition (SAI)

Several studies have demonstrated that this type of inhibition is reduced in individuals with MCI, especially in MCI due to AD.

Mimura et al. [46] showed that SAI, using TMS-EEG, is characterized by lower amplitude of N100 evoked potential (TMS-evoked potential, TEP) in MCI group compared to healthy controls. The study just cited, in addition to reporting a significant reduction in SAI, also highlights that by employing a different methodology—namely TMS-EEG—it is possible to obtain the same parameter that is traditionally derived from TMS-EMG, thereby underscoring the complementary role of these two approaches.

Two studies [47,55] reported reduced inhibition in individuals with aMCI; in particular, Nardone and colleagues observed that the multiple domain subgroup showed the most pronounced reduction of SAI compared to healthy subjects and other MCI subtypes.

Padovani and colleagues [51] reported greater SAI reduction in MCI due to AD (MCI-AD) than MCI non-AD subtypes. Benussi et al. [43] also found impaired SAI in MCI due to DLB (MCI-DLB) subtype.

Other authors have focused on different populations, observing altered SAI in idiopathic REM behavioral Disorder (iRBD) [48], in Parkinson’s disease (PD) patients with REM behavioral disorder (PD-RBD) [49], and in PD with MCI (PD-MCI) [56].

Some of the aforementioned authors have also reported correlations between SAI alterations and performance on neuropsychological tests. Nardone et al. [47] described a correlation between SAI and scores on the Digit Span, Trail Making Test parts A and B, the Stroop Color–Word Test, and various measures of the Rey Auditory Verbal Learning Test in aMCI. Two other studies [48,56] reported a correlation between altered inhibition and cognitive test scores. Nardone et al. [48] found correlations with measures of episodic verbal memory and executive function in iRBD; while Yarnall et al. [56] reported a correlation with the Montreal Cognitive Assessment scores in PD patients.

Additionally, Padovani and colleagues [51] found a positive correlation between SAI and levels of CSF t-tau and p-tau in MCI-AD.

However, three studies have not found any significant differences or alterations of SAI measure between MCI and HC [44,53,54].

### 3.3. Short-Intracortical Inhibition (SICI)

Only three works have reported significant findings indicating alterations in SICI in subjects with MCI. Padovani et al. [51] found impaired SICI in MCI non-AD compared to MCI-AD, and a correlation between SICI and CSF t-tau levels in both groups. Benussi and colleagues [43] observed impaired SICI in both MCI-FTD and MCI-DLB subtypes. Kamble et al. [45] reported significant impairment in patients with extrapyramidal symptoms, that increased progressively in PD patients to PD-MCI and PD with dementia (PDD) patients.

Five studies did not find any significant alterations in the SICI index [44,47,48,49,55] in MCI groups. Another study [50] showed a trend toward significance at a 2 ms interstimulus interval (ISI) between MCI-AD and HC.

### 3.4. Intracortical Facilitation (ICF)

Most of the studies [47,48,49,55] did not find any significant results concerning ICF.

However, Olazaran et al. [50] observed a trend of less facilitation in MCI-AD compared to HC.

Benussi and colleagues [43] found impaired ICF in both MCI-FTD and MCI-DLB subtypes, and Kamble [45] reported reduced facilitation as the disease progressed from PD through PD-MCI to PDD.

### 3.5. Long-Intracortical Inhibition (LICI)

Only two studies addressing LICI was identified in the literature search [43,50]; this index was not found to be impaired in any of the patient groups considered in Benussi or in Olazaran.

## 4. Discussion

Although MCI is a condition with a high incidence rate [7] and a risk factor for the development of dementia over the long term, there is a need of investigating early neurophysiological diagnostic markers.

TMS has emerged as a practical, safe, and efficient tool, not only in the research/scientific field, but also in the diagnostic domain, contributing to a better understanding of pathophysiology and providing real-time information [14].

While CSF and amyloid PET demonstrate high accuracy (85–95%) in distinguishing MCI-AD from MCI non-AD, TMS achieves comparable accuracy (91.3%) while offering a more non-invasive and cost-effective alternative [51].

While many studies have investigated the use of TMS in neurodegenerative diseases [14,19,20,34], studies in individuals with MCI remain limited and show heterogeneous results. Some studies have not found significant differences between MCI subjects and healthy controls, whereas others have reported neurophysiological alterations. However, comparing findings across studies is challenging due to the consideration of a considerable methodological variability which may have led to different results within the same population: different clinical populations have been included with variable etiological characterization, acquisition procedures vary, some studies distinguish between amnestic and non-amnestic MCI, while others treat MCI as a single entity. Additionally, in some cases, cognitive impairment has been investigated in the context of other neurodegenerative conditions, such as PD, further complicating the interpretation of the results.

Since MCI often progress to AD, most of the studies identified through the literature review on TMS indices have focused on SAI, with the specific purpose of investigating cholinergic impairment [27], not only through the use of TMS-EMG, but also by demonstrating that it is possible to employ a high–temporal resolution technique such as TMS-EEG to investigate the same phenomenon [46], thereby opening the possibility for future applications of this methodology in the study of other neurophysiological markers and cortical plasticity, especially in MCI.

Sakuma et al. [54] found impaired SAI in AD population, but not in individuals with aMCI or HC. However, a limit of this study, acknowledged by the authors themselves, was the absence of follow-up measures necessary to capture the critical point at which SAI becomes abnormal. On the contrary different authors found that patients with aMCI have greater SAI reduction compared to the other MCI subtypes [47,55]. This supports the hypothesis that SAI is an early marker in individuals at higher risk of developing AD. However, cholinergic dysfunction is a hallmark of Lewy bodies pathology. In this view, other studies reviewed here suggest that reduced SAI may also be associated Lewy bodies-related disorders, such as MCI with iRBD, MCI-DLB, or PDD [48,49,56]. Altogether, these findings suggest that this index could be helpful not only in the early diagnosis of AD, but also in patients with different pathologies characterized by cholinergic dysfunction. Accordingly, it has been demonstrated that treatment with acetylcholinesterase inhibitors, specifically donepezil, was able to restore this index [47].

Additionally, SICI and ICF were investigated by the majority of the articles considered. However, the results were less consistent compared to those observed for SAI. However, alterations in SICI and ICF were primarily observed in MCI non-AD [51] while individuals with MCI-AD showed more impaired SAI. It would have been interesting to know the etiological diagnoses within the MCI non-AD group, as this would allowed for a more precise characterization of the typical changes in these indices.

Other studies did not find any alterations in SICI and ICF in the MCI group [55], suggesting that these two indices may not be optimal markers for the diagnosis of early-stage AD, but could instead potentially be more relevant for other MCI phenotypes. Supporting this hypothesis, Benussi and colleagues [43] took into account a cohort of 106 MCI subjects, discriminated by their subtype, and found that impairments in both SICI and ICF allowed to distinguish MCI-FTD and MCI-DLB from other phenotypes.

Kamble et al. [45] analyzed cognitive impairment in PD patients and, contrary to previous studies [57,58], found significant impairments in SICI and ICF both in PD-MCI and in PDD. These results suggest that, in addition to cholinergic deficiency, progression from PD without cognitive decline through PD-MCI to PDD may involve enhanced GABAergic and reduced glutamatergic neurotransmission. Importantly, this highlights that TMS measures can differentiate Parkinsonian syndromes with cognitive impairment (PD-MCI, PDD) from those without dementia, reflecting distinct neurophysiological profiles across the spectrum of the disease.

The results reported above prompted a study where the authors analyzed TMS parameters recorded from 107 individuals with different MCI subtypes to evaluate whether these indices could enhance the clinicians’ diagnostic confidence of the clinical work-up alone. Indeed, clinicians’ diagnostic confidence significantly increased with the disclosure of TMS measures, highlighting the added value of TMS in differentiating MCI subtypes and in complementing current biomarker workflow. In this sense, TMS could serve not only as a supportive tool alongside established biomarkers, but also as a cost-effective and accessible screening method, potentially enabling earlier stratification and intervention in at-risk individuals.

Based on the considerations regarding the parameters examined and the related studies, it is also important to highlight the potential use of TMS through additional stimulation protocols, such as theta-burst stimulation (TBS) and paired associative stimulation (PAS). These protocols allow the investigation of other aspects of cortical activity with potential future clinical relevance in the field of dementia, including long-term potentiation (LTP) and long-term depression (LTD). Some studies [59,60] have demonstrated that the application of TMS can be extended beyond paired-pulse protocols, providing insights into cortical plasticity processes, which are often impaired in individuals with dementia. Expanding knowledge on these two protocols, particularly in the context of MCI would be a valuable objective, as they may contribute to a deeper understanding of the disorder and offer potential diagnostic applications.

## 5. Conclusions

This systematic review has several limitations. Due to the scarcity of studies in the existing literature, it would have been preferable not to restrict the search to only Title/Abstract fields, but to adopt a broader strategy. Nevertheless, this more focused approach enabled a highly specific selection and facilitated the identification of the most relevant studies. To mitigate the risk of missing relevant studies, we conducted the search across three databases.

A key finding of this review is the need for longitudinal studies and extended follow-up in the context of MCI, which, by definition, may progress into various neurodegenerative disorders.

A crucial step forward for TMS research and its potential clinical application is the standardization of experimental procedures to improve comparability across studies and ensure methodological rigor. In cases where full standardization is not feasible, reliable methods to normalize neurophysiological data should be implemented. Additionally, to enable TMS to serve effectively as a screening tool, it is necessary to establish standardized protocols, build normative datasets, and ensure that specialized personnel receive proper training. This highlights the practical steps needed to move from experimental use to clinical implementation. In conclusions, substantial work is still needed to integrate SAI, SICI and ICF indexes, and TMS in general, into routine clinical practice. Given the high conversion rate from MCI to AD and other neurodegenerative dementias, TMS could offer a practical and accessible screening tool to support timely decision-making and diagnostic algorithms. This is particularly important given the short time window often observed between MCI and dementia conversion [2,7]. EEG-informed TMS could also represent a valuable method to enhance the assessment of cortical function in these populations, as it allows real-time monitoring of neural activity, improves the signal-to-noise ratio of TMS-evoked potentials, and enables closed-loop stimulation tailored to individual cortical dynamics [61].

## Figures and Tables

**Table 1:** Literature review on TMS measures in mild cognitive impairment.

Publication	N	Gender, Female	Diagnosis	HC Group (Y = Yes; N = No)	Indices	Main Results
[54]	43	NA	AD, aMCI	Y	SAI	Impaired in AD vs. HC or aMCI
[50]	23	52.0%	MCI-AD	Y	SICI	No differences
					ICF	No differences
					LICI	No differences
[47]	50	36.0%	aMCI, na-MCI	Y	SAI	Impaired in aMCI vs. na-MCI or HC
					SICI	No differences
					ICF	No differences
[55]	28	64.2%	aMCI	Y	SAI	Impaired in aMCI vs. HC
					SICI	No differences
					ICF	No differences
[48]	25	NA	iRBD	Y	SAI	Impaired in iRBD vs. HC
					SICI	No differences
					ICF	No differences
[56]	44	45.5%	PD, PD-MCI	Y	SAI	Impaired in PD-MCI vs. PD or HC
[49]	38	26.3%	PD-RBD, PD-nRBD	Y	SAI	Impaired in PD-RBD vs. PD-nRBD or HC Abnormal in PD-RBD with MCI
					SICI	No differences
					ICF	No differences
[51]	69	58.0%	MCI-AD, MCI non-AD	Y	SAI	Impaired in MCI-AD vs. MCI non-AD or HC
					SICI	Impaired in MCI non-AD vs. MCI-AD or HC
					ICF	Impaired in MCI non-AD vs. MCI-AD or HC
[52]	107	50.0%	MCI-AD, MCI-FTD, MCI-DLB, MCI-other	N	SAI SICI ICF	Increased DC in MCI-AD, MCI-FTD, MCI-DLB, MCI-other
[43]	153	47.0%	MCI-AD, MCI-FTD, MCI-DLB	Y	SAI	Impaired in MCI-AD and MCI-DLB vs. HC
					SICI	Impaired in MCI-FTD and MCI-DLB vs. HC
					ICF	Impaired in MCI-FTD and MCI-DLB vs. HC
					LICI	No differences
[44]	30	40.0%	aMCI	Y	SAI	No differences
					SICI	No differences
[53]	56	62.5%	MCI	Y	SAI	No differences
[45]	76	19.7%	PD, PD-MCI, PDD	Y	SICI	Impaired in PDD, PD-MCI and PD vs. HC
					ICF	Impaired in PDD, PD-MCI and PD vs. HC
[46]	60	35.0%	MCI	Y	SAI	Impaired in MCI vs. HC

Abbreviations: AD, Alzheimer’s disease; aMCI, amnestic MCI; DC, diagnostic confidence; DLB, dementia with Lewy bodies; FTD, frontotemporal dementia; ICF, intracortical facilitation; iRBD, idiopathic RBD; LICI, long-interval intracortical inhibition; MCI, mild cognitive impairment; MCI-AD, MCI due to AD; MCI non-AD, MCI not due to AD; MCI-other, MCI due to other than AD/FTD/DLB; na-MCI, non-amnestic MCI; NA, not available; PD, Parkinson’s disease; PD-nRBD, PD without RBD; PDD, Parkinson’s disease dementia; REM behavioral disorder, RBD; SAI, short-latency afferent inhibition; SICI, short-interval intracortical inhibition.

## Data Availability

No new data were created or analyzed in this study.

## Supplementary Materials

The following supporting information can be downloaded at: https://www.mdpi.com/article/10.3390/brainsci15090969/s1, Figure S1: Risk-of-bias plot of included studies (adapted from [62]); Table S1: PRISMA 2020 for Abstracts Checklist [42]; Table S2: PRISMA 2020 Checklist [42].

## Abbreviations

The following abbreviations are used in this manuscript:

AD	Alzheimer’s disease
aMCI	amnestic mild cognitive impairment
CSF	cerebrospinal fluid
DC	diagnostic confidence
DLB	dementia with Lewy bodies
EEG	electromyography
EMG	electroencephalography
FTD	frontotemporal dementia
GABA	gamma-aminobutyric acid
HC	healthy control
ICF	intracortical facilitation
iRBD	idiopathic REM behavioral disorder
ISI	interstimulus interval
LICI	long interval intracortical inhibition
LTD	long-term depression
LTP	long-term potentiation
MCI	mild cognitive impairment
MCI-AD	mild cognitive impairment due to Alzheimer’s disease
MCI-DLB	mild cognitive impairment due to dementia with Lewy bodies
MCI-FTD	mild cognitive impairment due to frontotemporal dementia
MCI non-AD	mild cognitive impairment not due to Alzheimer’s disease
MCI-other	mild cognitive impairment due to other than AD/FTD/DLB
MD	multiple domain
na-MCI	non-amnestic mild cognitive impairment
PAS	paired associative stimulation
PD	Parkinson‘s disease
PD-MCI	Parkinson’s disease with mild cognitive impairment
PD-RBD	Parkinson’s disease with REM behavioral disorder
PDD	Parkinson‘s disease dementia
PET	positron emission tomography
PRISMA	Preferred Reporting Items for Systematic Reviews and Meta-Analyses
RBD	REM behavioral disorder
rTMS	repetitive transcranial magnetic stimulation
SAI	short-latency afferent inhibition
SD	single domain
SICI	short-interval intracortical inhibition
TBS	theta-burst stimulation
TEP	transcranial magnetic stimulation-evoked potential
TMS	transcranial magnetic stimulation
