# Supplementary material for: Transcranial Magnetic Stimulation as a Diagnostic Tool in Mild Cognitive Impairment: A Systematic Review
Source: Brain Sci. 2025 Sep 9;15(9):969. doi: 10.3390/brainsci15090969 (PMC12468978; doi:10.3390/brainsci15090969)
Supplement: Supplementary file 1 [file brainsci-15-00969-s001.zip › Figure S1.pdf]

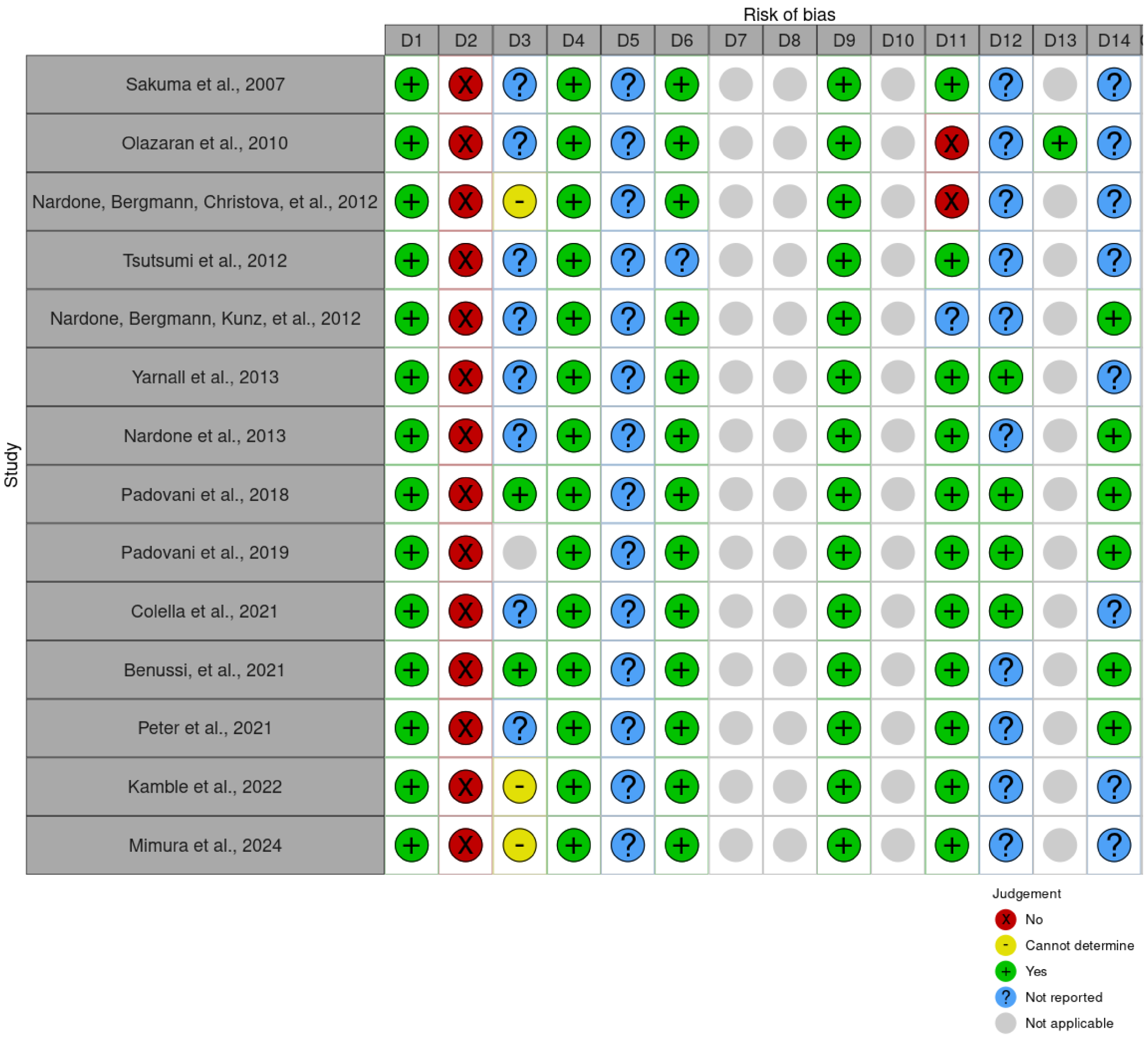

Figure S1: Risk-of-bias plot of included studies (adapted from McGuinness et al., 2020)

References

McGuinness, LA, Higgins, JPT. Risk-of-bias VISualization (robvis): An R package and Shiny web app for visualizing risk-of-bias assessments. *Res Syn Meth.* **2020**; 1- 7. https://doi.org/10.1002/jrsm.1411
