# Supplementary material for: Transcranial Magnetic Stimulation as a Diagnostic Tool in Mild Cognitive Impairment: A Systematic Review
Source: Brain Sci. 2025 Sep 9;15(9):969. doi: 10.3390/brainsci15090969 (PMC12468978; doi:10.3390/brainsci15090969)
Supplement: Supplementary file 1 [file brainsci-15-00969-s001.zip › Table S1.pdf]

PRISMA

PRISMA 2020 for Abstracts Checklist

Section and Topic	Item #	Checklist item	Reported (Yes/No)
**TITLE**			
Title	1	Transcranial Magnetic Stimulation as a diagnostic tool in Mild Cognitive Impairment: a systematic review	Yes
**BACKGROUND**			
Objectives	2	This systematic review aims to evaluate the diagnostic utility of TMS-derived indices, such as Short-latency Afferent Inhibition (SAI), Short-interval Intracortical Inhibition (SICI), Intracortical Facilitation (ICF), and Long-interval Intracortical Inhibition (LICI) in MCI populations	Yes
**METHODS**			
Eligibility criteria	3	Inclusion criteria: *(i)* original research; *(ii)* studies primarily focusing on at least one of the indices of interest (e.g., SAI, SICI, ICF, LICI); *(iii)* studies focusing on MCI or investigating MCI in the context of co-occurring neuro-degenerative disorders; and *(iv)* published before the 31/07/2025.<br>Exclusion criteria: *(i)* Studies focusing on MCI but that did not investigate any of the TMS indices of interest; *(ii)* Articles published in languages other than English; *(iii)* animal studies; *(iv)* reports of secondary data such as meta-analyses or reviews or letters.	Yes
Information sources	4	Following the PRISMA guidelines, we systematically searched PubMed, Embase and Scopus for articles published before the 31/07/2025	Yes
Risk of bias	5	All studies that met the inclusion criteria were assessed using the quality assessment 'Quality Assessment Tool for Observational Cohort and Cross-Sectional Studies' (https://www.nhlbi.nih.gov/health-topics/study-quality-assessment-tools).	Yes
Synthesis of results	6	All studies are summarized in a table (Results section)	Yes
**RESULTS**			
Included studies	7	14 full-text articles were included for this systematic review	Yes
Synthesis of results	8	Most studies report reduced SAI, a marker of cholinergic dysfunction, in amnestic MCI and MCI due to AD. Alterations in SICI and ICF, markers of GABAergic and glutamatergic dysfunction, were more variable, mainly observed in MCI of non-AD type. LICI showed no consistent changes. One study demonstrated increased clinicians' diagnostic confidence when TMS data were incorporated.	Yes
**DISCUSSION**			
Limitations of evidence	9	1) scarcity of studies in the existing literature<br>2) Lack of longitudinal studies and extended follow-up in the context of MCI<br>3) Inconsistency of experimental procedures across the studies	Yes
Interpretation	10	TMS measures hold promise as a non-invasive tool for early and differential diagnosis of MCI.	Yes
**OTHER**			
Funding	11	This research was funded by the European Union—Next Generation EU—PNRR M6C2—Investimento 2.1 Valorizzazione e potenziamento della ricerca biomedica del SSN (PNRR-MCNT2-2023-12377069;	Yes

Section and Topic	Item #	Checklist item	Reported (Yes/No)
		CUP=C83C24000200007).	
Registration	12	The protocol for this review was registered in the International Prospective Register of Systematic Reviews (PROSPERO) under registration number CRD420251112713	Yes

From: Page MJ, McKenzie JE, Bossuyt PM, Boutron I, Hoffmann TC, Mulrow CD, et al. The PRISMA 2020 statement: an updated guideline for reporting systematic reviews. BMJ 2021;372:n71. doi: 10.1136/bmj.n71. This work is licensed under CC BY 4.0. To view a copy of this license, visit https://creativecommons.org/licenses/by/4.0/
