# Supplementary material for: Transcranial Magnetic Stimulation as a Diagnostic Tool in Mild Cognitive Impairment: A Systematic Review
Source: Brain Sci. 2025 Sep 9;15(9):969. doi: 10.3390/brainsci15090969 (PMC12468978; doi:10.3390/brainsci15090969)
Supplement: Supplementary file 1 [file brainsci-15-00969-s001.zip › Table S2.pdf]

# PRISMA 2020 Checklist

| Section and Topic | Item # | Checklist item | Location where item is reported |
|---|---|---|---|
| **TITLE** | | | |
| Title | 1 | Identify the report as a systematic review. | Title Page |
| **ABSTRACT** | | | |
| Abstract | 2 | See the PRISMA 2020 for Abstracts checklist. | Structured abstract |
| **INTRODUCTION** | | | |
| Rationale | 3 | Describe the rationale for the review in the context of existing knowledge. | Introduction |
| Objectives | 4 | Provide an explicit statement of the objective(s) or question(s) the review addresses. | Introduction |
| **METHODS** | | | |
| Eligibility criteria | 5 | Specify the inclusion and exclusion criteria for the review and how studies were grouped for the syntheses. | Materials and Methods – Section *2.2 Study selection criteria* |
| Information sources | 6 | Specify all databases, registers, websites, organisations, reference lists and other sources searched or consulted to identify studies. Specify the date when each source was last searched or consulted. | Materials and Methods – Section *2.1 Search strategies and selection of the studies* |
| Search strategy | 7 | Present the full search strategies for all databases, registers and websites, including any filters and limits used. | Materials and Methods – Section *2.1 Search strategies and selection of the studies*<br>Section *2.2 Study selection criteria* |
| Selection process | 8 | Specify the methods used to decide whether a study met the inclusion criteria of the review, including how many reviewers screened each record and each report retrieved, whether they worked independently, and if applicable, details of automation tools used in the process. | Materials and Methods – Section *2.3 Data collection and extraction* Section *2.4 Quality assessment* |
| Data collection process | 9 | Specify the methods used to collect data from reports, including how many reviewers collected data from each report, whether they worked independently, any processes for obtaining or confirming data from study investigators, and if applicable, details of automation tools used in the process. | Materials and Methods – Section *2.3 Data collection and extraction* |
| Data items | 10a | List and define all outcomes for which data were sought. Specify whether all results that were compatible with each outcome domain in each study were sought (e.g. for all measures, time points, analyses), and if not, the methods used to decide which results to collect. | Introduction<br>Materials and Methods – Section *2.2 Study selection* |

| Section and Topic | Item # | Checklist item | Location where item is reported |
|---|---|---|---|
| | | | *criteria*<br>Materials and Methods – Section *2.3 Data collection and extraction* |
| | 10b | List and define all other variables for which data were sought (e.g. participant and intervention characteristics, funding sources). Describe any assumptions made about any missing or unclear information. | Materials and Methods – Section *2.2 Study selection criteria*<br>Materials and Methods – Section *2.3 Data collection and extraction* |
| Study risk of bias assessment | 11 | Specify the methods used to assess risk of bias in the included studies, including details of the tool(s) used, how many reviewers assessed each study and whether they worked independently, and if applicable, details of automation tools used in the process. | Materials and Methods – Section *2.4 Quality assessment* |
| Effect measures | 12 | Specify for each outcome the effect measure(s) (e.g. risk ratio, mean difference) used in the synthesis or presentation of results. | Not applicable (no effect size estimation) |
| Synthesis methods | 13a | Describe the processes used to decide which studies were eligible for each synthesis (e.g. tabulating the study intervention characteristics and comparing against the planned groups for each synthesis (item #5)). | Materials and Methods – Section *2.3 Data collection and extraction* |
| | 13b | Describe any methods required to prepare the data for presentation or synthesis, such as handling of missing summary statistics, or data conversions. | Materials and Methods – Section *2.3 Data collection and extraction* Results – Section *3.1 Study characteristics* |
| | 13c | Describe any methods used to tabulate or visually display results of individual studies and syntheses. | Results – Section *3.1 Study characteristics* and Table 1 |
| | 13d | Describe any methods used to synthesize results and provide a rationale for the choice(s). If meta-analysis was performed, describe the model(s), method(s) to identify the presence and extent of statistical heterogeneity, and software package(s) used. | A meta-analysis was not performed. |
| | 13e | Describe any methods used to explore possible causes of heterogeneity among study results (e.g. subgroup analysis, meta-regression). | Not applicable. No meta- analysis was conducted; therefore, no exploration of |

| Section and Topic | Item # | Checklist item | Location where item is reported |
|---|---|---|---|
| | | | statistical heterogeneity was performed. |
| | 13f | Describe any sensitivity analyses conducted to assess robustness of the synthesized results. | Not applicable. No quantitative synthesis or sensitivity analyses were conducted. |
| Reporting bias assessment | 14 | Describe any methods used to assess risk of bias due to missing results in a synthesis (arising from reporting biases). | Materials and Methods – Section *2.4 Quality assessment*<br>Supplementary Materials – Figure S2 |
| Certainty assessment | 15 | Describe any methods used to assess certainty (or confidence) in the body of evidence for an outcome. | A formal certainty assessment method such as GRADE was not applied. |
| **RESULTS** | | | |
| Study selection | 16a | Describe the results of the search and selection process, from the number of records identified in the search to the number of studies included in the review, ideally using a flow diagram. | Materials and Methods – Figure 1<br>Results – Section *3.1 Study characteristics* |
| | 16b | Cite studies that might appear to meet the inclusion criteria, but which were excluded, and explain why they were excluded. | Results – Section *3.1 Study characteristics* |
| Study characteristics | 17 | Cite each included study and present its characteristics. | Results –Table 1 |
| Risk of bias in studies | 18 | Present assessments of risk of bias for each included study. | Materials and Methods – Section *2.4 Quality assessment*<br>Supplementary Materials – Figure S2 |
| Results of individual studies | 19 | For all outcomes, present, for each study: (a) summary statistics for each group (where appropriate) and (b) an effect estimate and its precision (e.g. confidence/credible interval), ideally using structured tables or plots. | Not Applicable |
| Results of | 20a | For each synthesis, briefly summarise the characteristics and risk of bias among contributing studies. | Results – Section |

# PRISMA 2020 Checklist

| Section and Topic | Item # | Checklist item | Location where item is reported |
|---|---|---|---|
| syntheses | | | 3.2, 3.3, 3.4, 3.5<br>Risk of bias was qualitatively assessed. |
| | 20b | Present results of all statistical syntheses conducted. If meta-analysis was done, present for each the summary estimate and its precision (e.g. confidence/credible interval) and measures of statistical heterogeneity. If comparing groups, describe the direction of the effect. | Not applicable. No statistical synthesis or meta-analysis was conducted |
| | 20c | Present results of all investigations of possible causes of heterogeneity among study results. | Not Applicable |
| | 20d | Present results of all sensitivity analyses conducted to assess the robustness of the synthesized results. | Not applicable. No investigations of heterogeneity were conducted, as no statistical synthesis |
| Reporting biases | 21 | Present assessments of risk of bias due to missing results (arising from reporting biases) for each synthesis assessed. | No formal analysis of reporting bias was conducted. |
| Certainty of evidence | 22 | Present assessments of certainty (or confidence) in the body of evidence for each outcome assessed. | No formal assessment tool such as GRADE was used. However, the overall quality and consistency of the evidence were discussed qualitatively based on methodological rigor, and data reporting across studies. |
| **DISCUSSION** | | | |
| Discussion | 23a | Provide a general interpretation of the results in the context of other evidence. | The results were interpreted in light of prior research and in light of the heterogeneous results |
| | 23b | Discuss any limitations of the evidence included in the review. | Conclusion |
| | 23c | Discuss any limitations of the review processes used. | Conclusion |
| | 23d | Discuss implications of the results for practice, policy, and future research. | Discussion and |

PRISMA

# PRISMA 2020 Checklist

| Section and Topic | Item # | Checklist item | Location where item is reported |
|---|---|---|---|
| | | | Conclusion |
| **OTHER INFORMATION** | | | |
| Registration and protocol | 24a | Provide registration information for the review, including register name and registration number, or state that the review was not registered. | The protocol for this review was registered in the International Prospective Register of Systematic Reviews (PROSPERO) under registration number CRD420251112713 |
| | 24b | Indicate where the review protocol can be accessed, or state that a protocol was not prepared. | No review protocol was prepared or made publicly available. |
| | 24c | Describe and explain any amendments to information provided at registration or in the protocol. | Not Applicable |
| Support | 25 | Describe sources of financial or non-financial support for the review, and the role of the funders or sponsors in the review. | **Funding** section |
| Competing interests | 26 | Declare any competing interests of review authors. | **Conflict of Interest** section |
| Availability of data, code and other materials | 27 | Report which of the following are publicly available and where they can be found: template data collection forms; data extracted from included studies; data used for all analyses; analytic code; any other materials used in the review. | **Supplementary Materials** section |

*From:* Page MJ, McKenzie JE, Bossuyt PM, Boutron I, Hoffmann TC, Mulrow CD, et al. The PRISMA 2020 statement: an updated guideline for reporting systematic reviews. BMJ 2021;372:n71. doi: 10.1136/bmj.n71.
This work is licensed under CC BY 4.0. To view a copy of this license, visit https://creativecommons.org/licenses/by/4.0/
